# Legal and Ethical Consideration in Artificial Intelligence in Healthcare: Who Takes Responsibility?

**DOI:** 10.3389/fsurg.2022.862322

**Published:** 2022-03-14

**Authors:** Nithesh Naik, B. M. Zeeshan Hameed, Dasharathraj K. Shetty, Dishant Swain, Milap Shah, Rahul Paul, Kaivalya Aggarwal, Sufyan Ibrahim, Vathsala Patil, Komal Smriti, Suyog Shetty, Bhavan Prasad Rai, Piotr Chlosta, Bhaskar K. Somani

**Affiliations:** ^1^Department of Mechanical and Manufacturing Engineering, Manipal Institute of Technology, Manipal Academy of Higher Education, Manipal, India; ^2^International Training and Research in Uro-Oncology and Endourology Group, Manipal, India; ^3^Department of Urology, Father Muller Medical College, Mangalore, India; ^4^Department of Humanities and Management, Manipal Institute of Technology, Manipal Academy of Higher Education, Manipal, India; ^5^Department of Instrumentation and Control Engineering, Manipal Institute of Technology, Manipal Academy of Higher Education, Manipal, India; ^6^Robotics and Urooncology, Max Hospital and Max Institute of Cancer Care, New Delhi, India; ^7^Department of Radiation Oncology, Massachusetts General Hospital and Harvard Medical School, Boston, MA, United States; ^8^Department of Computer Science and Engineering, Manipal Institute of Technology, Manipal Academy of Higher Education, Manipal, India; ^9^Kasturba Medical College, Manipal Academy of Higher Education, Manipal, India; ^10^Department of Oral Medicine and Radiology, Manipal College of Dental Sciences, Manipal, Manipal Academy of Higher Education, Manipal, India; ^11^Department of Urology, Freeman Hospital, Newcastle upon Tyne, United Kingdom; ^12^Department of Urology, Jagiellonian University in Krakow, Kraków, Poland; ^13^Department of Urology, University Hospital Southampton National Health Service (NHS) Trust, Southampton, United Kingdom

**Keywords:** artificial intelligence, machine learning, ethical issues, legal issues, social issues

## Abstract

The legal and ethical issues that confront society due to Artificial Intelligence (AI) include privacy and surveillance, bias or discrimination, and potentially the philosophical challenge is the role of human judgment. Concerns about newer digital technologies becoming a new source of inaccuracy and data breaches have arisen as a result of its use. Mistakes in the procedure or protocol in the field of healthcare can have devastating consequences for the patient who is the victim of the error. Because patients come into contact with physicians at moments in their lives when they are most vulnerable, it is crucial to remember this. Currently, there are no well-defined regulations in place to address the legal and ethical issues that may arise due to the use of artificial intelligence in healthcare settings. This review attempts to address these pertinent issues highlighting the need for algorithmic transparency, privacy, and protection of all the beneficiaries involved and cybersecurity of associated vulnerabilities.

## Introduction

Increasing patient demand, chronic disease, and resource constraints put pressure on healthcare systems. Simultaneously, the usage of digital health technologies is rising, there has been an expansion of data in all healthcare settings. If properly harnessed, healthcare practitioners could focus on the causes of illness and keep track of the success of preventative measures and interventions. As a result, policymakers, legislators, and other decision-makers should be aware of this. For this to happen, computer and data scientists and clinical entrepreneurs argue that one of the most critical aspects of healthcare reform will be artificial intelligence (AI), especially machine learning (1). Artificial intelligence (AI) is a term used in computing to describe a computer program's capacity to execute tasks associated with human intelligence, such as reasoning and learning. It also includes processes such as adaptation, sensory understanding, and interaction. Traditional computational algorithms, simply expressed, are software programmes that follow a set of rules and consistently do the same task, such as an electronic calculator: “if this is the input, then this is the output.” On the other hand, an AI system learns the rules (function) through training data (input) exposure. AI has the potential to change healthcare by producing new and essential insights from the vast amount of digital data created during healthcare delivery (2).

AI is typically implemented as a system comprised of both software and hardware. From a software standpoint, AI is mainly concerned with algorithms. An artificial neural network (ANN) is a conceptual framework for developing AI algorithms. It's a human brain model made up of an interconnected network of neurons connected by weighted communication channels. AI uses various algorithms to find complex non-linear correlations in massive datasets (analytics). Machines learn by correcting minor algorithmic errors (training), thereby boosting prediction model accuracy (confidence) (3, 4).

The use of new technology raises concerns about the possibility that it will become a new source of inaccuracy and data breach. In the high-risk area of healthcare, mistakes can have severe consequences for the patient who is the victim of this error. This is critical to remember since patients come into contact with clinicians at times in their lives when they are most vulnerable (5). If harnessed effectively, such AI-clinician cooperation can be effective, wherein AI is used to offer evidence-based management and provides medical decision-guide to the clinician (AI-Health). It can provide healthcare offerings in diagnosis, drug discovery, epidemiology, personalized care, and operational efficiency. However, as Ngiam and Khor point out if AI solutions are to be integrated into medical practice, a sound governance framework is required to protect humans from harm, including harm resulting from unethical behavior (6–17). Ethical standards in remedy may be traced lower back to the ones of the health practitioner Hippocrates, on which the idea of the Hippocratic Oath is rooted (18–24).

Machine Learning-healthcare applications (ML-HCAs) that were seen as a tantalizing future possibility has become a present clinical reality after the Food and Drug Administration (FDA) approval for autonomous artificial intelligence diagnostic system based on Machine Learning (ML). These systems use algorithms to learn from large data sets and make predictions without explicitly programming (25).

## Applications of AI for Health Research

The use of data created for electronic health records (EHR) is an important field of AI-based health research. Such data may be difficult to use if the underlying information technology system and database do not prevent the spread of heterogeneous or low-quality data. Nonetheless, AI in electronic health records can be used for scientific study, quality improvement, and clinical care optimization. Before going down the typical path of scientific publishing, guideline formation, and clinical support tools, AI that is correctly created and trained with enough data can help uncover clinical best practices from electronic health records. By analyzing clinical practice trends acquired from electronic health data, AI can also assist in developing new clinical practice models of healthcare delivery (26).

## Artificial Intelligence in Drug Development

In the future, AI is expected to simplify and accelerate pharmaceutical development. AI can convert drug discovery from a labor-intensive to capital- and the data-intensive process by utilizing robotics and models of genetic targets, drugs, organs, diseases and their progression, pharmacokinetics, safety and efficacy. Artificial intelligence (AI) can be used in the drug discovery and development process to speed up and make it more cost-effective and efficient. Although like with any drug study, identifying a lead molecule does not guarantee the development of a safe and successful therapy, AI was used to identify potential Ebola virus medicines previously (26).

## Ethical Challenges

There is a continuous debate regarding whether AI “fits within existing legal categories or whether a new category with its special features and implications should be developed.” The application of AI in clinical practice has enormous promise to improve healthcare, but it also poses ethical issues that we must now address. To fully achieve the potential of AI in healthcare, four major ethical issues must be addressed: (1) informed consent to use data, (2) safety and transparency, (3) algorithmic fairness and biases, and (4) data privacy are all important factors to consider (27). Whether AI systems may be considered legal is not only a legal one but also a politically contentious one (Resolution of the European Parliament, 16 February 2017) (28).

The aim is to help policymakers ensure that the moral demanding situations raised by enforcing AI in healthcare settings are tackled proactively (17). The limitation of algorithmic transparency is a concern that has dominated most legal discussions on artificial intelligence. The rise of AI in high-risk situations has increased the requirement for accountable, equitable, and transparent AI design and governance. The accessibility and comprehensibility of information are the two most important aspects of transparency. Information about the functionality of algorithms is frequently deliberately made difficult to obtain (29).

Our capacity to trace culpability back to the maker or operator is allegedly threatened by machines that can operate by unfixed rules and learn new patterns of behavior. The supposed “ever-widening” divide is a cause for alarm, as it threatens “both the moral framework of society and the foundation of the liability idea in law.” The use of AI may leave us without anyone to hold accountable for any sort of damage done. The extent of danger is unknown, and the use of machines will severely limit our ability to assign blame and take ownership of the decision-making (30).

Modern computing approaches can hide the thinking behind the output of an Artificial Intelligent System (AIS), making meaningful scrutiny impossible. Therefore, the technique through which an AIS generates its outputs is “opaque.” A procedure used by an AIS may be so sophisticated that for a non-technically trained clinical user, it is effectively concealed while remaining straightforward to understand for a techie skilled in that area of computer science (5).

AISs, like IBM's Watson for oncology, are meant to support clinical users and hence directly influence clinical decision-making. The AIS would then evaluate the information and recommend the patient's care. The use of AI to assist clinicians in the future could change clinical decision-making and, if adopted, create new stakeholder dynamics. The future scenario of employing AIS to help clinicians could revolutionize clinical decision-making and, if embraced, create a new healthcare paradigm. Clinicians (including doctors, nurses, and other health professionals) have a stake in the safe roll-out of new technologies in the clinical setting (5).

The scope of emerging ML-HCAs in terms of what they intend to achieve, how they might be built, and where they might be used is very broad. ML-HCAs range from entirely self-sufficient synthetic intelligence diabetic retinopathy prognosis in primary care settings, to non-self-sufficient death forecasts, to manual coverage and resource allocation (25). Researchers ought to describe how those outputs can be included in the research, along with predictions. This information is essential to setting up the cost of the scientific trial and guiding scientific research (31).

AI applied in healthcare needs to adjust to a continuously changing environment with frequent disruptions, while maintaining ethical principles to ensure the well-being of patients (24). However, an easy, key component of figuring out the protection of any healthcare software relies upon the capacity to check out the software and recognize how the software would fail. For example, the additives and physiologic mechanisms of medications or mechanical devices are comparable to the technique for software programmes. On the other hand, ML-HCAs can present a “black box” issue, with workings that aren't visible to evaluators, doctors, or patients. Researchers ought to describe how those outputs can be included in the research, along with predictions. This information helps assess the cost of the scientific trial and guides scientific research (25).

## Global Legislations

**Figure 1:**
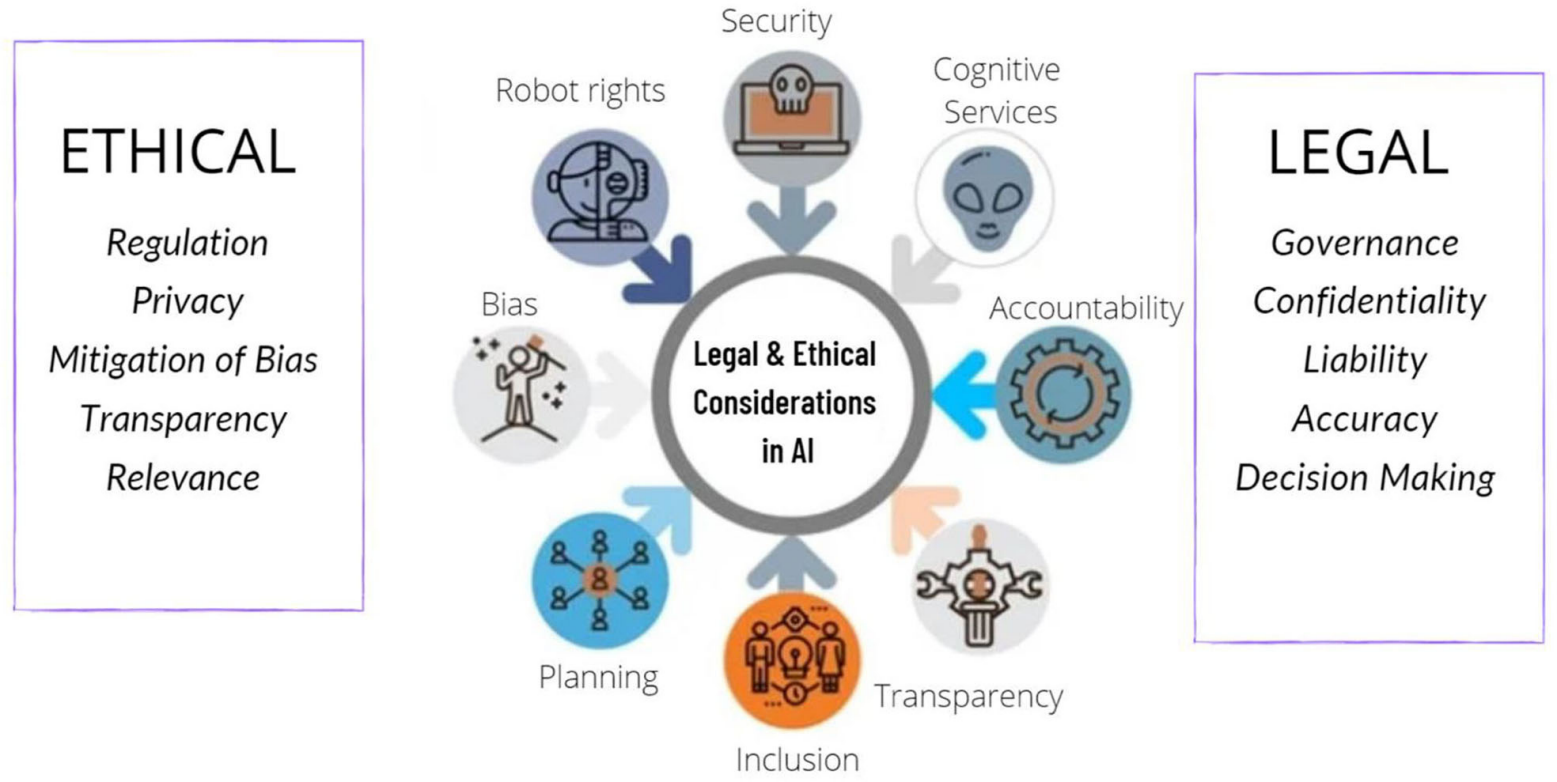
Various ethical and legal conundrums involved with the usage of artificial intelligence in healthcare.

The Resolution of the European Parliament was based on research commissioned, supervised, and published by the policy department for “Citizens' Rights and Constitutional Affairs” in response to a request from the European Parliament's Committee on Legal Affairs. The report emphasizes the critical nature of a resolution calling for the immediate creation of a legislative instrument governing robots and AI, capable of anticipating and adapting to any scientific breakthroughs anticipated in the medium term (29). The various ethical and legal concerns associated with the use of AI in healthcare settings have been highlighted in Figure 1.

## Why is Responsibility Necessary?

When the setting or context changes, AI systems can fail unexpectedly and drastically. AI can go from being extremely intelligent to extremely naive in an instant. All AI systems will have limits, even if AI bias is managed. The human decision-maker must be aware of the system's limitations, and the system must be designed so that it fits the demands of the human decision-maker. When a medical diagnostic and treatment system is mostly accurate, medical practitioners who use it may grow complacent, failing to maintain their skills or take pleasure in their work. Furthermore, people may accept decision-support system results without questioning their limits. This sort of failure will be repeated in other areas, such as criminal justice, where judges have modified their decisions based on risk assessments later revealed to be inaccurate (32).

The use of AI without human mediation raises concerns about vulnerabilities in cyber security. According to a RAND perspectives report, applying AI for surveillance or cyber security in national security creates a new attack vector based on “data diet” vulnerabilities. The study also discusses domestic security issues, such as governments' (growing) employment of artificial agents for citizen surveillance (e.g., predictive policing algorithms). These have been highlighted as potentially jeopardizing citizens' fundamental rights. These are serious concerns because they put key infrastructures at risk, putting lives and human security and resource access at risk. Cyber security weaknesses can be a severe threat because they are typically hidden and only discovered after the event (after the damage is caused) (28).

In recent years, there has been an uptick in the feasibility, design, and ethics of lethal autonomous weapon systems (LAWS). These machines would have AI autonomy's vast discretion combined with the power to murder and inflict damage on humans. While these advancements may offer considerable advantages, various questions have been raised concerning the morality of developing and implementing LAWS (33).

The problem of selection bias in datasets used to construct AI algorithms is a typical occurrence. As established by Buolamwini and Gebru, there is bias in automated facial recognition and the associated datasets, resulting in lower accuracy in recognizing darker-skinned individuals, particularly women. A huge number of data points are required for ML, and the majority of frequently utilized clinical trial research databases come from selected populations. As a result, when applied to underserved and consequently probably underrepresented patient groups, the resulting algorithms may be more likely to fail (34).

## Who Bears the Responsibility?

Unlike doctors, technologists are not obligated by law to be accountable for their actions; instead, ethical principles of practice are applied in this sector. This comparison summarizes the dispute over whether technologists should be held accountable if AIS is used in a healthcare context and directly affects patients. If a clinician can't account for the output of the AIS they're employing, they won't be able to appropriately justify their actions if they choose to use that data. This lack of accountability raises concerns about the possible safety consequences of using unverified or unvalidated AISs in clinical settings. Some scenarios show how opacity affects each stakeholder. Table 1 shows the necessary considerations for procedural and conceptual changes to be taken for ethical review for healthcare-based Machine learning research. It is indeed a challenging aspect of technology. We think that new framework and approach is needed for approval of AI systems but practitioners and hospitals using it need to be trained and hence have the ultimate responsibility of its use. Medical devices based AI will facilitate the decision making too carry out treatment and procedures by the individuals, and not to replace them in entirety. There is dearth of literature in this regard and a detailed frame-work needs to be developed by the highest bodies of policy makers.

**Table 1:** Considerations for ethical review for healthcare-based Machine learning research: procedural and conceptual changes (31).

**Stage 1: Data access**	
Group-based approval	Providing access to specific, qualified individuals who are grouped around a common governance structure, subject to certain conditions, and with a specific aim in mind.
PHI (Protected health information) protection	PHI that isn't required is deleted, leaving the option of examining raw or masked data.
Broad goal without pre-determined methodology	Allows comparison of alternative methodologies to help implementation and avoids biasing study outputs.
Data-access frameworks	A greater emphasis on data governance, with accountability gained by access and rationale records.
Pre-specified, frequent data retrieval without repeated amendments	Ascertains if the model is learning from the most recent patterns in health data.
**Stage 2: Silent period**	
Prospective non-interventional trial application as a template	Patients do not receive treatments, and machine learning results do not reach the treating team in time to influence decision-making or the trial's evaluation.
Goal of the trial	To see if the model is feasible and if it can be used in clinical settings.
Model validation	Technical performance and calibration were evaluated using ML best practices.
Clinical evaluation	By comparing quiet predictions to real-time patient labeling, evidence for the model's clinical usefulness is obtained.
**Stage 3: Clinical trial**	
Goal of the trial	To see if the model is more effective than the existing standard of treatment.
Generalizability	Rather than demonstrating the model's generalizability, the goal is to demonstrate the approach's generalizability.
Disaggregated performance metrics	Patient safety and justice depend on disaggregated performance indicators, which will guide clinical acceptance.
Clinically relevant evaluation	Disaggregated performance measures will guide clinician acceptance, ensuring patient safety and justice. • The model was investigated in the context of its planned application in decision-making. • The outputs of the model were recorded. • Clinical decisions are kept track of. •Determining the cause of a disparity in output and decisions.

AISs should be evaluated and validated, according to the Association for the Advancement of Artificial Intelligence. It is critical to establish, test, measure, and assess the dependability, performance, safety, and ethical compliance of such robots and artificial intelligence systems logically and statistically/probabilistically before they are implemented. If a clinician chooses to employ an AIS, verification and validation may help them account for their activities reasonably. As previously mentioned, clinical rules of professional conduct do not allow for unaccountable behavior. It has been suggested, however, that AIS is not the only thing that may be opaque, and doctors can also be opaque. If AIS cannot be punished, it will be unable to take on jobs involving human care. Managers of AIS users should make it clear that physicians cannot evade accountability by blaming the AIS (5).

Assistive ML-HCAs provide resources to healthcare providers by providing “ideas” for treatment, prognosis, or control while relying on individual interpretation of any suggestions to make judgments. Autonomous ML-HCAs provide direct prognostic and control statements without the intervention of a clinician or any other human. Because the developer's preference for an ML-autonomy HCA's stage has clear implications for the assumption of responsibility and liability, this autonomy stage must be visible (25). Instead of asking if they were aware of the hazards and poor decision-making, the question should be asked if they could grasp and recognize those risks (35).

## Bias in the Use of AI

Evidence suggests that AI models can embed and deploy human and social biases at scale. However, it is the underlying data than the algorithm itself that is to be held responsible. Models can be trained on data which contains human decisions or on data that reflects the second-order effects of social or historical inequities. Additionally, the way data is collected and used can also contribute to bias and user-generated data can act as a feedback loop, causing bias. To our knowledge there are no guidelines or set standards to report and compare these models, but future work should involve this to guide researchers and clinicians (36, 37).

AI is moving beyond “nice-to-have” to becoming an essential part of modern digital systems. As we rely more and more on AI for decision making, it becomes absolutely essential to ensure that they are made ethically and free from unjust biases. We see a need for Responsible AI systems that are transparent, explainable, and accountable. AI systems increase in use for improving patient pathways and surgical outcomes, thereby outperforming humans in some fields. It is likely to meager, co-exist or replace current systems, starting the healthcare age of artificial intelligence and not using AI is possibly unscientific and unethical (38).

## Conclusion

AI is going to be increasingly used in healthcare and hence needs to be morally accountable. Data bias needs to be avoided by using appropriate algorithms based on un-biased real time data. Diverse and inclusive programming groups and frequent audits of the algorithm, including its implementation in a system, need to be carried out. While AI may not be able to completely replace clinical judgment, it can help clinicians make better decisions. If there is a lack of medical competence in a context with limited resources, AI could be utilized to conduct screening and evaluation. In contrast to human decision making, all AI judgments, even the quickest, are systematic since algorithms are involved. As a result, even if activities don't have legal repercussions (because efficient legal frameworks haven't been developed yet), they always lead to accountability, not by the machine, but by the people who built it and the people who utilize it. While there are moral dielemmas in the use of AI, it is likely to meager, co-exist or replace current systems, starting the healthcare age of artificial intelligence, and not using AI is also possibly unscientific and unethical.

## Author Contributions

NN, DSh, BH, and BS contributed to the conception and design of the study. MS, SI, DSw, SS, and KA organized the database. DSw, KA, VP, KS, SS, and SI wrote the first draft of the manuscript. NN, DSh, KS, BR, VP, and BH wrote sections of the manuscript. PC, BR, and BS critically reviewed and edited the manuscript. All authors contributed to manuscript revision, read, and approved the submitted version.

## Conflict of Interest

The authors declare that the research was conducted in the absence of any commercial or financial relationships that could be construed as a potential conflict of interest.

## Publisher's Note

All claims expressed in this article are solely those of the authors and do not necessarily represent those of their affiliated organizations, or those of the publisher, the editors and the reviewers. Any product that may be evaluated in this article, or claim that may be made by its manufacturer, is not guaranteed or endorsed by the publisher.
